# UNC-45A Is Highly Expressed in the Proliferative Cells of the Mouse Genital Tract and in the Microtubule-Rich Areas of the Mouse Nervous System

**DOI:** 10.3390/cells10071604

**Published:** 2021-06-26

**Authors:** Valentino Clemente, Asumi Hoshino, Joyce Meints, Mihir Shetty, Tim Starr, Michael Lee, Martina Bazzaro

**Affiliations:** 1 Masonic Cancer Center and Department of Obstetrics, Gynecology and Women’s Health, University of Minnesota, Minneapolis, MN 55455, USA; vclement@umn.edu (V.C.); hoshi020@umn.edu (A.H.); shett036@umn.edu (M.S.); star0044@umn.edu (T.S.); 2 Department of Neuroscience, University of Minnesota, Minneapolis, MN 55455, USA; meint002@umn.edu (J.M.); mklee@umn.edu (M.L.)

**Keywords:** UNC-45A, ovaries, neurons, brain, cilia, microtubules

## Abstract

UNC-45A (Protein unc-45 homolog A) is a cytoskeletal-associated protein with a dual and non-mutually exclusive role as a regulator of the actomyosin system and a Microtubule (MT)-destabilizing protein, which is overexpressed in human cancers including in ovarian cancer patients resistant to the MT-stabilizing drug paclitaxel. Mapping of UNC-45A in the mouse upper genital tract and central nervous system reveals its enrichment not only in highly proliferating and prone to remodeling cells, but also in microtubule-rich areas, of the ovaries and the nervous system, respectively. In both apparatuses, UNC-45A is also abundantly expressed in the ciliated epithelium. As regulators of actomyosin contractility and MT stability are essential for the physiopathology of the female reproductive tract and of neuronal development, our findings suggest that UNC-45A may have a role in ovarian cancer initiation and development as well as in neurodegeneration.

## 1. Introduction

UNC-45A (Protein unc-45 homolog A) is a member of the UCS (UNC-45/CRO1/She4p) protein family [1,2], with dual and non-mutually exclusive roles as a regulator of the actomyosin system [3,4] and a microtubule (MT)-destabilizing protein [5,6,7]. Regulators of actomyosin contractility and MT stability are essential for both ovarian cancer cell proliferation and neuronal development. For instance, dysregulation of the Rho/ROCK (Rho-associated protein kinase) signaling pathway is commonly found in ovarian cancer [8,9,10,11] and implicated in the pathophysiology of the nervous system [12,13]. Several MT-destabilizing proteins are also expressed in both neurons [14,15,16,17,18] and cancer cells, including ovarian cancer cells [19,20,21], where they play roles spanning from regulating symmetrical and asymmetrical cell division [22] to regulating MT mass [6] and sensitivity to MT-targeting agents [6,23]. As a key regulator of cytoskeletal activities, UNC-45A participates in several cellular functions including cytokinesis [24,25,26,27], exocytosis [28], and axonal growth [29]. UNC-45A is overexpressed in breast and ovarian cancer as compared to their normal counterpart [24,25,26] and in ovarian cancer patients that are resistant to the MT-stabilizing drug paclitaxel [6].

In light of the role played by UNC-45A in regulating cell proliferation and of its role as a MAP with MT-destabilizing properties, the objective of this study was to determine the pattern of UNC-45A expression in cells of the mouse genital tract with different proliferative and remodeling capacities and in the mouse central nervous system (CNS).

We found that UNC-45A is enriched in the highly proliferating and prone to remodeling cells of the mouse reproductive system, in the microtubule-rich regions of the mouse central nervous system and in the cilia of both, the mouse reproductive system and the brain. Taken together, these findings suggest that UNC-45A may play a role in the physiology and pathology of the female reproductive apparatus and the central nervous system.

## 2. Materials and Methods

### 2.1. Sample Preparation of Mouse CNS and Upper Genital Tract

Ovaries, fallopian tubes, brains, and spinal cords from adult C57BL/6 mice were preserved in 10% formalin buffer solution for 24 h followed by 70% ethanol to prevent the tissue from decomposing, and embedded in paraffin. At least two different mice were used to evaluate the UNC-45A expression level in CNS and mouse upper genital tract. The phase of the estrous cycle was determined by histological evaluation as previously described [30,31].

### 2.2. Antibodies

Anti-UNC-45A Rabbit Polyclonal antibody (19564-1-AP, Protein Tech, Rosemont, IL, USA) was used for IHC and IF. The specificity of this antibody has been previously validated by us and others by Western Blot, IF, and IHC analyses [5,6]. The Anti-NeuN mouse monoclonal antibody (MA5-33103, ThermoFisher, Waltham, MA, USA) was used for IF. Secondary antibodies for IF analyses were FITC-conjugated Goat Anti-Rabbit IgG used at 1:200 dilution (Jackson ImmunoResearch Laboratories, West Grove, PA, USA) and Texas-Red conjugated Goat Anti-Mouse IgG used at 1:200 dilution (Jackson ImmunoResearch Laboratories, West Grove, PA, USA).

### 2.3. Immunohistochemical Staining of Mouse Upper Genital Tract, Brain, and Spinal Cord

Five μm formalin-fixed; paraffin-embedded (FFPE) tissue sections were subjected to hematoxylin and eosin staining and immunohistochemistry (IHC) for UNC-45A. Slides were deparaffinized with 100% xylene and rehydrated with gradient ethanol (100%, 95%, and 80%). Antigen retrieval was then carried out with 1X Reveal Decloaker (Biocare Medical, Pacheco, CA, USA) in a vegetable steamer for 30 min at 100 °C, before blocking the slides with Background Sniper (BS966H, Biocare Medical, Pacheco, CA, USA) for 13 min at room temperature. Sections were subsequently incubated with anti-UNC45A antibody at a dilution of 1:100 for mouse brain and spinal cord and 1:200 for mouse upper genital tract overnight at 4 °C, followed by Biotin-SP-conjugated AffiniPure Goat Anti-Rabbit IgG (111-065-003, Jackson ImmunoResearch Laboratories, West Grove, PA, USA) at a dilution of 1:200 for 30 min at room temperature and incubation with horseradish peroxidase streptavidin at a dilution of 1:125 (405210, BioLegend, San Diego, CA, USA) for 30 min at room temperature. After the staining was developed with 3,3′-diaminobenzidine (926506, BioLegend, San Diego, CA, USA) for 3 min, the slides were counterstained with Harris’ hematoxylin.

### 2.4. Bright-Field Imaging

Brightfield images were acquired with a Zeiss Axio Scan.Z1 system (Zeiss, Thornwood, NY, USA) at 40× magnification. Images of details were taken with a BX40 light microscope, DP72 camera, and cellSens Standard v1.16 imaging software (Olympus, Waltham, MA, USA).

### 2.5. Immunofluorescence (IF) Microscopy

For immunofluorescence analysis of UNC-45A and NeuN (neuronal nuclear protein) in a mouse brain, 5 μm formalin-fixed; paraffin embedded (FFPE) tissue sections were deparaffinized, rehydrated, retrieved, and blocked similarly to what described in the immunohistochemical staining section. Sections were then incubated with anti-UNC45A and anti-NeuN at the indicated concentration overnight at 4 °C. Secondary antibodies were used at the indicated concentration for 1 h at room temperature and sections were mounted with mounting media containing DAPI (4′,6-diamidino-2-phenylindole) (F6057 Sigma-Aldrich. Burlington, MA, USA). Images were obtained on an Axiovert 200 microscope (Zeiss, Thornwood, NY, USA) equipped with a high-resolution CCD camera.

### 2.6. Quantification of UNC-45A Expression Levels via Immunofluorescence and via Immunohistochemistry

Quantification of immunohistochemistry was done on ImageJ using a slightly modified version of a previously described protocol [32]. Briefly, using whole slide scansions, regions of interest (ROIs) were drawn around the borders of the single elements analyzed before saving them into the ROI manager. Images were then converted into their RGB format and colors deconvolved using the color deconvolution function of ImageJ. Finally, the DAB signal was thresholded with a fixed upper limit of 70, and the mean grey values representing pixel intensity were obtained from individual ROIs. For quantification of immunofluorescence, pixel intensity was measured from the green channel after drawing the ROIs, and the background was subtracted without further image processing. At least three different areas were measured for each histological element and data compared using GraphPad Prism (version 8.4.3, San Diego, CA, USA).

## 3. Results

### 3.1. UNC-45A Is Expressed in the Mouse Upper Genital Tract with a Stronger Expression in Proliferating Cells and Cilia

**Figure 1:**
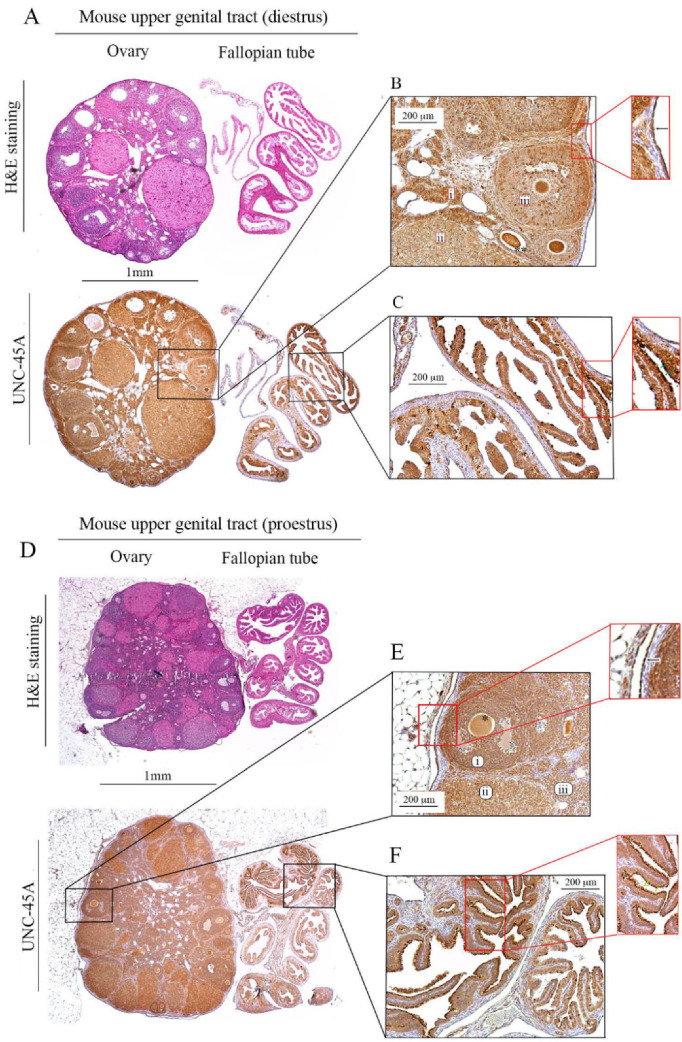
UNC-45A (Protein unc-45 homolog A) expression in mouse ovaries and fallopian tube during diestrus and proestrus. (**A**). H&E (hematoxylin and eosin) (upper panel) and UNC-45A (lower panel) staining of the mouse ovary and fallopian tube during diestrus (**B**). Details of mouse ovary with follicular structures strongly stained for UNC-45A (i. early follicles, ii. corpus luteum, and iii. later follicles) and containing oocytes (asterisks). Inset, mouse ovarian surface epithelium (arrow) positive for UNC-45A while the stroma is negative. (**C**). Details of mouse fallopian tube with epithelium strongly stained for UNC-45A. Inset, cilia in fallopian tube epithelium (green arrows) strongly positive for UNC-45A. (**D**). H&E (upper panel) and UNC-45A (lower panel) staining of the mouse ovary and fallopian tube during proestrus (**E**). Details of mouse ovary with follicular structures strongly stained for UNC-45A (i. later follicles, ii. corpus luteum, and iii. early follicles) and containing oocytes (asterisks). Inset, mouse ovarian surface epithelium (arrow) positive for UNC-45A while the stroma is negative. (**F**). Details of mouse fallopian tube with epithelium strongly stained for UNC-45A. Inset, cilia in fallopian tube epithelium (green arrows) strongly positive for UNC-45A.

We and others have previously shown that UNC-45A is expressed in human ovarian surface epithelial cells and human breast cells and overexpressed in highly proliferative cells of ovarian and breast cancers as compared to their normal counterpart [24,25]. Here, we performed H&E (hematoxylin and eosin) and UNC-45A staining (Figure 1A, upper and lower panels, respectively) in mouse ovaries and fallopian tubes (diestrus) to determine UNC-45A expression pattern in the mouse upper genital tract during diestrus. We found that UNC-45A is abundantly expressed in cells of the early follicles (Figure 1B, i), the corpus luteum (Figure 1B, ii), and the later follicles (Figure 1B, iii).

**Figure 2:**
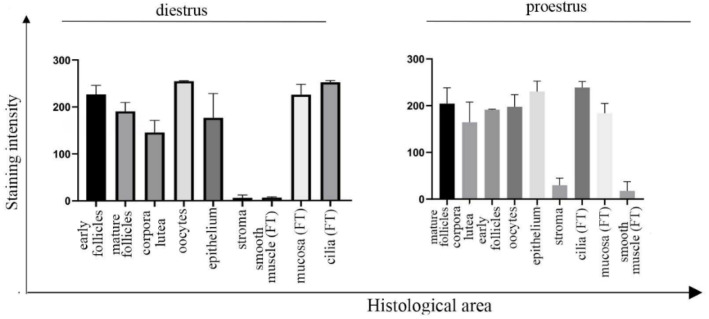
Quantification of UNC-45A expression levels in mouse ovaries and fallopian tubes (FTs) during diestrus and proestrus following IHC staining. Quantification of UNC-45A staining intensity in the single components of the ovary and fallopian tube. Quantification is expressed as mean pixel intensity, during diestrus (**left**) and proestrus (**right**).

Furthermore, UNC-45A is strongly expressed in the oocytes (Figure 1B black asterisks) and the ovarian surface epithelium (Figure 1B, inset, black arrow), while the stroma is mostly negative. In the fallopian tubes, UNC-45A is weakly expressed in the smooth muscle cells and more intensively expressed in the fallopian tube epithelium and in particular in the cilia of the fallopian tube epithelium (Figure 1C and its inset, green arrows). Quantification of UNC-45A levels in the ovary and fallopian tube during diestrus as determined by immunohistochemistry are given in Figure 2 (left). Importantly, cilia are microtubule (MT)-based structures capable of moving themselves using dynein contractility, and we have recently shown that UNC-45A is a Microtubule-Associated-Protein (MAP) responsible for modulating MT dynamics [7,33,34]. Taken together, this suggests that in the mouse upper genital tract UNC-45A expression is stronger in highly proliferative and prone to remodeling cells and within that UNC-45A may play a role in regulating cilia functions.

We also performed H&E and UNC-45A staining of the mouse ovary and fallopian tube during proestrus. This phase was chosen because, while the follicles of short cycling animals like mice are constantly proliferating, the element of the mouse ovary mostly affected by significant changes in proliferation and activity during different phases is represented by the corpus luteum [30,31], which degenerates during proestrus. We found no drastic changes in the UNC-45A expression pattern, with all the previous structures being strongly stained by UNC-45A (Figure 1D and its insets). Of note, as compared to the diestrus, more mature follicles displayed a slightly more intense staining than their earlier counterparts (Figure 1E, i and iii). Corpora lutea, which are degenerating prevalently in this phase, displayed slightly decreased expression of UNC-45A (Figure 1E, ii). No changes were observed in the surface epithelium (Figure 1E and its inset, black arrow), the fallopian tube (Figure 1F and its inset) and the cilia (Figure 1F and its inset, green arrows). Quantification of UNC-45A levels in the ovary and fallopian tube during proestrus as determined by immunohistochemistry are given in Figure 2 (right).

### 3.2. UNC-45A Is Expressed in the Microtubule-Rich Regions of the Mouse Central Nervous System (CNS) and Mouse Nerve Roots

**Figure 3:**
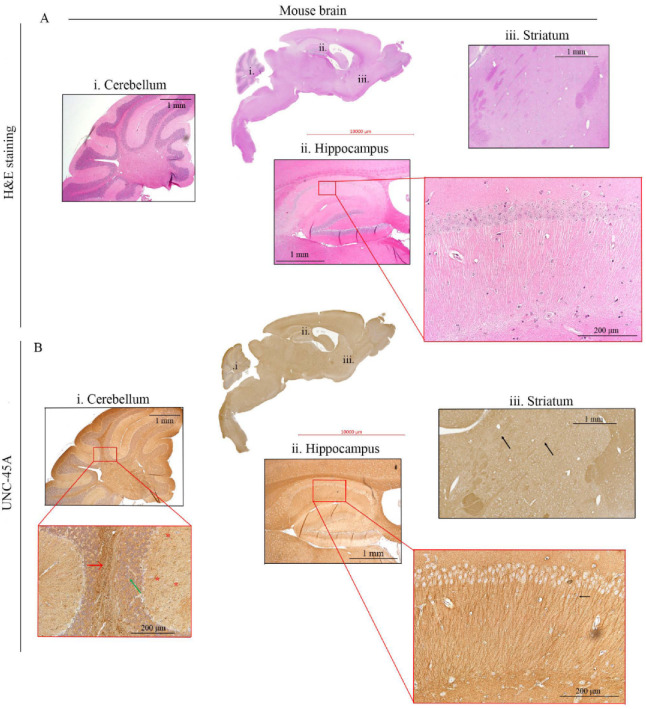
UNC-45A expression in mouse brain via IHC. (**A**). H&E staining of the whole mouse brain. i. Cerebellum, ii. Hippocampus, iii. Striatum. (**B**). UNC-45A staining in the whole mouse brain. i. details on UNC-45A staining in Cerebellum (inset) from the center to the sides: white matter (red arrow) granular layer (green arrow), molecular layer (red asterisks), ii. details of UNC-45A staining in hippocampus, inset closer view on hippocampal CA1 neurons, with clearly visible dendrites (black arrow). iii. details of UNC-45A staining in the striatum, arrows indicate stronger UNC-45A staining in nervous bundles.

We have recently shown that UNC-45A is a Microtubule-Associated-Protein (MAP) with MT-severing properties [5,6,7], which is important for neurite development [29]. In neurons, MTs play a pivotal role in both the structure and the function of axons and dendrites, whereas MAPs have been extensively shown to be major regulators of the physiology and pathology of the nervous system [35,36]. Here, we performed H&E and UNC-45A staining (Figure 3A,B, respectively) of the whole adult mouse brain. H&E staining allowed for visualization of brain anatomical structures including the cerebellum (Figure 3A, i), the hippocampus (Figure 3A, ii and its inset), and the striatum (Figure 3A, iii).

**Figure 4:**
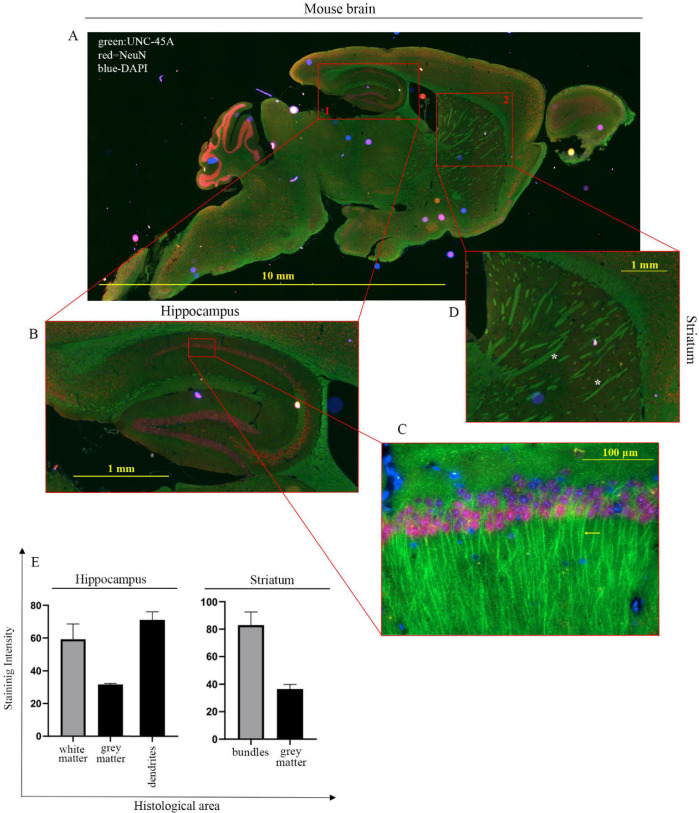
UNC-45A expression in mouse brain via immunofluorescence (IF). (**A**). Immunofluorescence staining for UNC-45A (green), NeuN (neuronal nuclear protein—magenta), and DAPI (4′,6-diamidino-2-phenylindole—blue) in the whole mouse brain. (**B**). Details of the stained hippocampus. (**C**). Close-up of UNC-45A staining in dendrites of hippocampal neurons (yellow arrow), DAPI staining of nuclei, and NeuN staining the neuronal soma. (**D**). Details of UNC-45A staining in the striatum (white asterisks indicate the nervous bundles). (**E**). Quantification of expression levels of UNC-45A in the indicated histological areas of the CNS. Quantification is expressed as mean pixel intensity.

We found that in the cerebellum UNC-45A is most strongly expressed in the white matter (Figure 3B, i, and its inset, red arrow), while the intensity of the staining decreases progressively in the granular (Figure 3B, i, and its inset, green arrow) and the molecular (Figure 3B, i, and its inset, red asterisks) layers of the cortex. This difference in UNC-45A staining intensity between the two cortical structures might be explained by their peculiar architecture: the granular layer is formed by neuronal bodies intercalated to the axonal fibers descending into the white matter, while the molecular layer is formed by a sparse network of dendrites, some of which stand out from the background (Figure 3B, i, next to the asterisks) due to their high UNC-45A expression. We also found that UNC-45A is abundantly expressed in the dendrites of the CA1 hippocampal neurons (Figure 3B, ii. and its inset, black arrow). In the striatum, UNC-45A is strongly expressed in the nervous bundles formed by axons crossing this grey structure (Figure 3B, iii, black arrows). To gain more details on the pattern of UNC-45A expression in the mouse brain, we performed immunofluorescence analysis on the whole mouse brain stained for UNC-45A (green), the neuronal marker NeuN [37] (magenta), and DAPI (blue) to stain nuclei (Figure 4A). As shown in Figure 4B,C, in hippocampal CA1 neurons, UNC-45A has a perinuclear and cytoplasmic localization and is particularly enriched in the dendrites (yellow arrow). Furthermore, UNC-45A was also confirmed to have strong staining in the nervous bundles of the striatum (Figure 4D, white asterisks). Quantification of UNC-45A expression levels in the brain as determined via immunofluorescence is given in Figure 4E.

**Figure 5:**
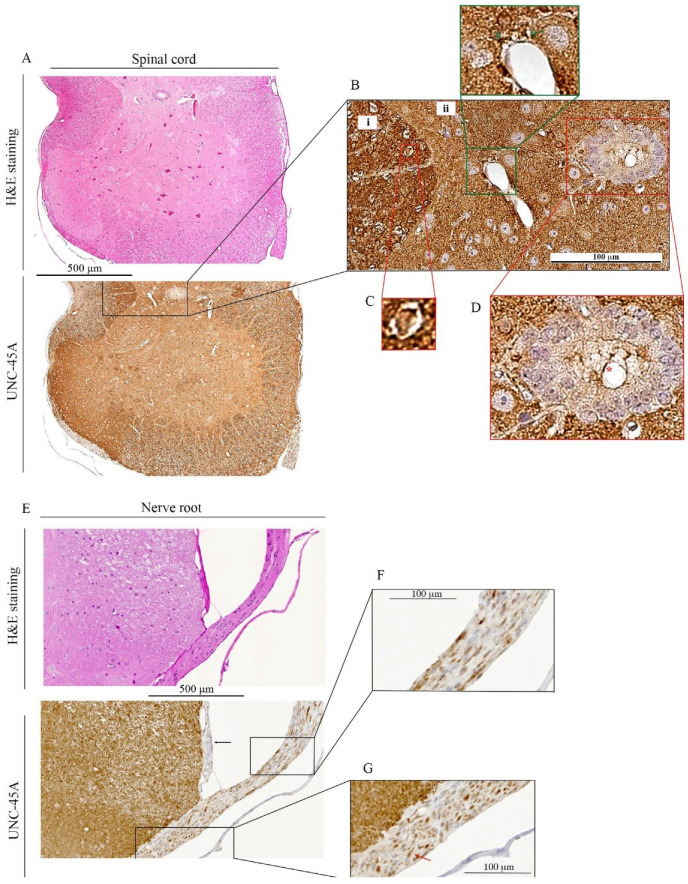
UNC-45A expression in mouse spinal cord and nerve root. (**A**). H&E (upper panel) and UNC-45A (lower panel) staining of the mouse spinal cord. (**B**). Details of UNC-45A staining in white matter (i), grey matter (ii), perivascular area (green arrow). (**C**). Detail of UNC-45A staining of a single cross-sectioned axon with its myelin coat. (**D**). UNC-45A staining in ependymocytes and their ciliary structures (red asterisk). (**E**). H&E (upper panel) and UNC-45A (lower panel) staining of the mouse nerve root (black arrow indicates the meninge). (**F**). UNC-45A staining in the longitudinal section of the nerve root. (**G**). UNC-45A staining in cross-section of the nerve root (red arrow indicates the myelin sheath).

**Figure 6:**
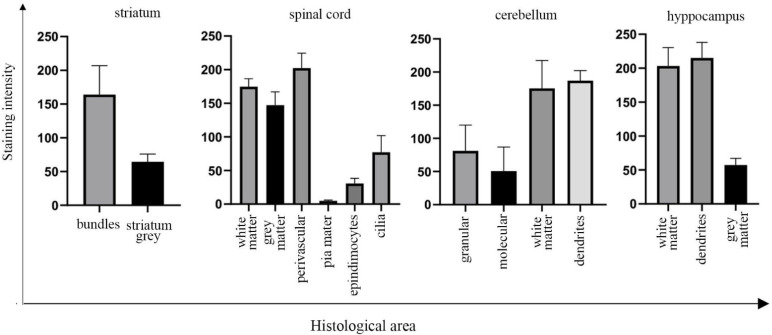
Quantification of UNC-45A expression levels in the mouse central nervous system (CNS) following IHC staining. Quantification of UNC-45A staining intensity in the indicated histological areas of the CNS following IHC staining. Quantification is expressed as mean pixel intensity.

Next, we performed H&E and UNC-45A staining of the mouse spinal cord (Figure 5A upper and lower panels, respectively) and mouse nerve root (Figure 5E, upper and lower panel, respectively). In the spinal cord, UNC-45A is more abundantly expressed in the white matter (Figure 5B, i.) than in the grey matter (Figure 5B, ii). In the white matter, intense UNC-45A staining can be seen in cross-sections of single axons (Figure 5C), surrounded by UNC-45A-negative myeline sheath. Interestingly, a strong UNC-45A expression can also be found in the feet of the astrocytes forming the hematoencephalic barrier in the area around vessels (Figure 5B, green inset, green arrows) while the ependymocytes express UNC-45A prevalently in their cilia (Figure 5D, red asterisk), with the cytoplasm being just weakly positive. In the nerve root, the meninges (Figure 5E lower panel, black arrow) are negative for UNC-45A while a strong UNC-45A staining can be found in both, longitudinal (Figure 5F) and cross-sections (Figure 5G). In the cross-section of the nerve roots, it is evident how strongly UNC-45A positive axons are surrounded by a negatively stained myelin sheath (red arrow). Quantification of UNC-45A expression levels in the mouse CNS as determined via immunohistochemistry is given in Figure 6.

Taken together, this suggests that UNC-45A may play a relevant role in nervous system physiology. In particular, considering the crucial roles of MTs and their MAPs in neuronal biology [35,36], the preferential and abundant expression of UNC-45A in axons and dendrites of mature neurons shows that this protein is not only required for their development [29], but may also be important for the neuronal functions and homeostasis. This could thus hint at a role for UNC-45A in more complex processes such as brain plasticity and/or neurodegeneration.

## 4. Discussion

Here, we found that in the mouse upper genital tract UNC-45A is enriched in proliferating and prone to remodeling cells as compared to quiescent cells. When we took into account possible variabilities in the expression of UNC-45A following the hormonal and structural changes that accompany the progression of the estrous cycle, we found no significant differences in UNC-45A expression pattern in diestrus vs. proestrus. Given that the transition from diestrus to proestrus occurs with an important hormonal shift from progesterone to estrogens, these findings suggest that UNC-45A expression could be only indirectly related to female hormones. On the other hand, considering UNC-45A known roles, minor changes may more likely be explained by the differences in proliferation that naturally occur during phase transition. Indeed, if compared to diestrus, during proestrus we found higher levels of UNC-45A in the more mature follicles, which are also more proliferative in this phase, but smaller differences were found in the corpus luteum, where UNC-45A may be still required for the cytoskeletal remodeling that occurs during cellular degeneration. Of note is that we found that UNC-45A is strongly expressed in both the oocyte and the ciliated epithelium, which plays a crucial role during fertility, as it allows for the progression of the oocyte along the fallopian tube after ovulation. These findings, along with the fact that UNC-45A is overexpressed in ovarian cancer [25] and that MT-severing proteins are involved in oocyte development [38,39,40], hint at the potential relevance of UNC-45A in the pathophysiology of the ovaries including reproductive biology and ovarian cancer initiation. In addition, our ex vivo observation that UNC-45A is highly expressed in proliferative cells adds strength to the importance of UNC-45A in the proliferation at the cellular level. This is consistent with previous reports [5,24,25,26,27] and, especially in ovarian cancer, UNC-45A is necessary to support the growth of cancer cells and to develop chemoresistance to paclitaxel [5,6,25]. The latter is a distinctive trait of recurrent ovarian cancer [41] and is often thought to arise from undifferentiated cancer cells (generally referred to as cancer stem-like cells) [42] and, particularly, UNC-45A expression has been inversely correlated with differentiation [24,25,43].

We also found that UNC-45A is expressed in neurons in the mouse nervous system and is particularly enriched in areas containing axons and dendrites. Interestingly, Tau, a MAP that stabilizes MT via preventing MT severing [44], is largely expressed in the brain, its hyperphosphorylation is associated with Alzheimer’s disease (AD) and other tauopathies [45,46,47,48,49,50], and it is abnormally expressed in ovarian cancer [51,52]. Importantly, drugs targeting MT stability are an established anti-cancer approach for ovarian cancer [53,54] and stabilization of neuronal MTs can attenuate neurodegenerative in mouse models of AD and other tauopathies [55]. In this context, our results suggest that UNC-45A may play a role in the physiology of the central nervous system as well as in neurodegeneration.

Lastly, we found that UNC-45A is abundantly expressed in both the ciliated columnar epithelium of the fallopian tube and the cilia of the ependymocytes. Interestingly, congenital loss-of-function mutations in *UNC-45A* cause a syndrome characterized by diarrhea, cholestasis, bone fragility, and impaired hearing [56], all of which symptoms involve organs where cilia play important roles in physiology [57,58,59]. Furthermore, almost all of the subjects affected by congenital UNC-45A loss have signs of intellectual disability [56]. Although this may be partially due to deafness or/and other aspecific and indirect causes associated with the syndrome, it is also possible that UNC-45A loss may be directly responsible for the intellectual disability of these patients given its wide expression in the central nervous system and its requirement for the extension of neurites [29]. Taken together, our results also suggest that UNC-45A may play a role in the physiology of ciliated epithelia as well as in ciliopathies.
